# The Incidence and Effect of Cytomegalovirus Disease on Mortality in Transplant Recipients and General Population: Real-world Nationwide Cohort Data

**DOI:** 10.7150/ijms.62621

**Published:** 2021-07-25

**Authors:** Sang Hoon Han, Seul Gi Yoo, Kyung Do Han, Yeonju La, Da Eun Kwon, Kyoung Hwa Lee

**Affiliations:** 1 Divison of Infectious Disease, Department of Internal Medicine, Yonsei University College of Medicine, Seoul, Republic of Korea; 2 Department of Statistics and Actuarial Science, Soongsil University, Seoul, Republic of Korea

**Keywords:** Cytomegalovirus, Disease, Incidence, Mortality, Population, Solid organ transplantation, Hematopoietic stem cell transplantation

## Abstract

**Background**: In addition to the conventional opportunistic infections in solid organ transplantation (SOT) and hematopoietic stem cell transplantation (HSCT) recipients, cytomegalovirus (CMV) infection is associated with various chronic inflammatory diseases or poor outcomes in non-immunocompromised critically ill patients. To evaluate the burden or outcome of CMV replication in non-transplant individuals, we compared the incidence rates (IRs) for CMV disease and all-cause mortality between SOT recipients, HSCT recipients, and non-transplant population.

**Methods**: The SOT (N=16,368) and HSCT (N=10,206) cohorts between 2010 and 2015 were established using the WHO ICD-10 from the whole population-based large database of the Health Insurance Review & Assessment Service (HIRA). CMV cases, defined as symptomatic disease with isolation of virus, DNA, pp65 antigen, and pathology except CMV syndrome, were extracted with the unique codes for relief of medical costs of HIRA in the same dataset. Cox's proportional hazard regression analyses and log-rank test in the Kaplan-Meier curves were performed to compare all-cause mortality between the three groups.

**Results**: The CMV IRs adjusted by age and sex were significantly higher in the SOT (adjusted IR [95% confidence intervals], 33.1 [28.8-38.0] per 1,000 person-years) and HSCT recipients (5.1 [4.6-6.1] per 1,000 person-years) than in the whole population (0.58 [0.49-0.67] per 100,000 person-years). However, SOT recipients with CMV (18/283, 6.4%) had significantly lower all-cause mortality than non-transplant individuals with CMV (207/1,258, 16.5%) (adjusted hazard ratio [95% CI], 0.42 [0.25-0.67], log-rank *P* < 0.001).

**Conclusion**: These data suggest that CMV disease in patients without transplants is associated with poor outcomes.

## Introduction

The ubiquitous cytomegalovirus (CMV) can lead to diverse clinical outcomes through acute direct or chronic indirect pathogenesis in populations with different comorbidities 1-3. In the era of growing aging population and immunomodulation drugs, CMV could exert an influence on public health in various ways, including chronic inflammatory vascular disorders besides tissue-invasive end-organ disease 1. Recently, several studies have reported the association of CMV with neurocognitive function, metabolic diseases, stroke, myocardial infarction, autoimmune diseases, or frailty in addition to well-known conventional opportunistic infections in severely immunocompromised patients or congenital CMV infection 4-13. The non-transplant, non-immunocompromised, critically ill patients in intensive care unit are another risk group, who have a higher rate of CMV reactivation on mortality (approximately 15-20% of incidence), even though it is not obvious whether active CMV replication has a causal relationship with life-threatening outcomes or represents the bystander effect by hyperactivation of immune system in severe diseases 14-17. Taken together, the burden of potential CMV infection or disease caused by life-long periodic reactivation necessitates the development of CMV vaccine for widespread elimination 18-20.

Despite the clinical significance and impact, the precise incidence rate (IR) per person-years of CMV infection or disease in solid organ transplantation (SOT) or hematopoietic stem cell transplantation (HSCT) recipients as well as non-transplant individuals has not been evaluated 1. Most studies assessing post-SOT CMV epidemiology presented only the frequency (percentage) of CMV infection or disease in a single institution with relatively small recipients (6.2-60%, 1-65%, 19-72.1%, and 15-33.9% in kidney, liver, heart, and pancreas transplantation, respectively) in clinical settings applying various preventive strategies against CMV 21-31. Similarly, the overall incidence of CMV infection or disease after allo-HSCT was reported to range from 3% to 40% 32-37. In addition, the definition of CMV infection or disease as well as the cut-off threshold for clinically significant DNAemia were not uniform in publications 21-23,27,28,30,32. Some studies defined CMV infection as asymptomatic viremia, DNAemia, or antigenemia, while others used suspected or probable end-organ disease 22,23,32,38. The most recent systemic review of real-world data in kidney transplant recipients showed that the estimated pooled incidences of CMV infection, including acute viral syndrome or end-organ disease, were quite heterogeneous according to the various risk factors, severity of immunosuppression, or methods of CMV prevention, including the dosage of anti-CMV agents 39.

The association between CMV and various comorbidities or mortality in general population has been evaluated previously using CMV seropositivity rates or immunoglobulin G (IgG) concentrations, but do not demonstrate the presence of virus in the host body as shown by methods, such as the measurement of CMV DNA, virion, or pp65 antigen 12,13,40-43. However, the clear definition of CMV disease should be based on virus isolation by culture, nucleic acid amplification, or protein tests 30,44. The result of CMV IgG concentration may represent the past exposure history and/or the current status of humoral immunity, but does not indicate active lytic CMV replication 30,44,45.

Therefore, we assessed the difference in IRs for CMV per person-years between SOT recipients, HSCT recipients, and non-transplant individuals and the effect of CMV end-organ disease except CMV syndrome on all-cause mortality in these groups to evaluate the clinical impact and burden of active CMV replication, defined as CMV detection by polymerase chain reaction, culture, shell viral assay, antigen test, or histopathology, using a general population-based large cohort dataset.

## Materials and methods

### Cohort construction and data extraction

The Korean National Health Insurance System (NHIS) organized by the government provides a single healthcare insurance service to the entire nation, covering approximately 50 million people. The Health Insurance Review & Assessment Service (HIRA), managed by the NHIS, establishes the whole database consisting of general information, diagnosis, drug, and healthcare services subset to operate the efficient claim process to healthcare providers 46. Using the nationwide database warehouse in the HIRA, we constructed SOT and HSCT recipient cohorts who received transplantation between January 2010 and December 2015. For the retrieval of recipients, we used the unique HIRA codes (V084, V085, V086, V087, V088 for SOT, and V081, V082, V083 for HSCT) to identify the rare incurable diseases for the direct relief co-payment policy 47, which are perfectly matched with the International Statistical Classification of Disease and Related Health Problems 10^th^ Revision (ICD-10) codes version 2016 by the World Health Organization (Z94.0, Z94.1, Z.94.2, Z.94.3, Z.94.4, and Z94.8) 48. The SOT and HSCT cohorts for 6 years included 16,368 and 10,206 recipients, respectively.

The HIRA includes CMV disease except CMV syndrome as rare incurable diseases and manages a strict review process for the relief of medical costs 47. For application of the unique code V104 for CMV disease except CMV syndrome, physicians should submit the results of CMV detection in blood, body fluid, or tissues using histopathologic findings, nucleic amplification test of polymerase chain reaction, pp65 antigen test, virus culture, and shell viral assay to the HIRA. The definition of CMV disease in the HIRA process is matched by the ICD-10 codes of B25 (cytomegaloviral disease), B25.0 (cytomegaloviral pneumonitis), B25.1 (cytomegaloviral hepatitis), B25.2 (cytomegaloviral pancreatitis), B25.8 (other cytomegaloviral diseases), and B25.9 (cytomegaloviral disease, unspecified), and does not include the P35.1 (congenital CMV infection) or B27.1 (cytomegaloviral mononucleosis) 48. The asymptomatic CMV isolation as CMV viremia, DNAemia, and pp65 antigenemia in the regular screening or surveillance for post-transplant CMV prevention is not acceptable to submission for the HIRA review. We used the term 'CMV cases' in this study, defined as CMV disease except CMV syndrome 30,44.

The co-morbid diseases were identified by ICD-10 codes: (1) type 2 diabetes mellitus (DM) (E11), (2) end stage renal disease (ESRD) on dialysis (N18.5), (3) chronic heart diseases including congestive heart failure (CHF) (I50.0), left ventricular failure (I50.1), heart failure, unspecified (I50.9), myocardial infarction (I21, I22, I25.2), hypertensive heart disease with CHF (I11.0), and cardiomyopathy (I42, I25.5), (4) chronic liver diseases including hepatic failure (K72, K70.4), chronic viral hepatitis (B18.0, B18.1, B18.2, B18.8, B18.9), and liver cirrhosis (K74, K70.3), (5) chronic lung diseases including chronic obstructive pulmonary diseases (J44), chronic emphysema (J43.8, J43.9), and interstitial lung diseases (J84), (6) human immunodeficiency virus-1 (HIV-1) infection (B20-B24, Z21).

This study was approved by the NHIS and the Gangnam Severance Institutional Review Board, and informed consent was waived because of the anonymous data (Study No: REQ0000017664 and IRB No: 3-2017-0341).

### Statistical analyses

The data are expressed as numbers (percentage) or means ± standard deviations, as well as IRs or hazard ratios (HRs) (95% confidence intervals [CI]). The standardized IRs and HRs per person-years were obtained by adjusting for age and sex using the 2010 South Korea Population and Housing Census data. We used the ANOVA and chi-square test to compare the continuous and nominal variables, respectively, between the three groups. Cox proportional hazards regression analysis was performed to compare the mortality rates between SOT recipients, HSCT recipients, and non-transplant individuals. Kaplan-Meier curves were constructed to compare the incidence probability of CMV cases or all-cause death. All statistical analyses were performed using SAS software (version 9.1; SAS Institute, Cary, NC, USA), and a two-tailed P-value of < 0.05, was considered statistically significant.

## Results

### Incidence rates of CMV cases in SOT and HSCT recipients

**Figure 1:**
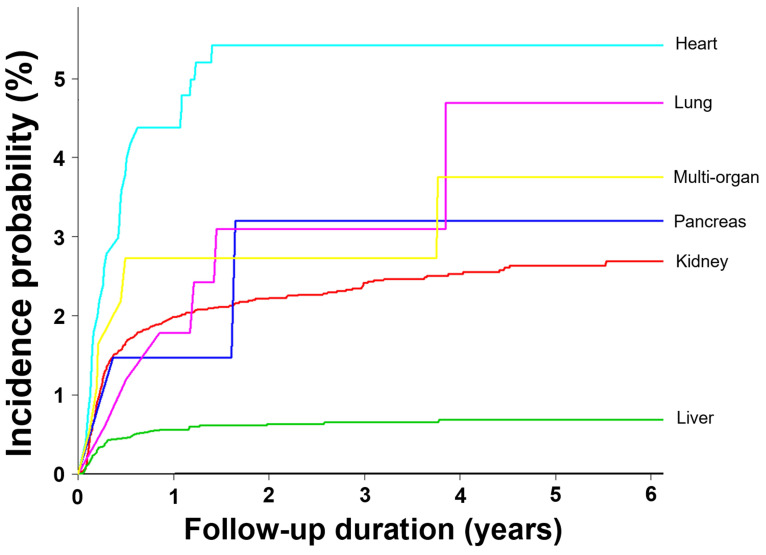
Difference in incidence probability for development of CMV disease except CMV syndrome according to the transplant organ in solid organ transplantation recipients

The percentages of patients with CMV cases were 0.003%, 1.7%, and 0.44% in non-transplant individuals, SOT recipients, and HSCT recipients, respectively. The adjusted IR of CMV in the whole-population cohort, including 50 million individuals per year between 2010 and 2015 was 0.58/100,000 person-years (Table 1). The SOT (adjusted IR [95% CI], 33.1 [28.8-38.0] per 1,000 person-years) and HSCT recipients (5.1 [4.6-6.1] per 1,000 person-years) had significantly higher IRs than that in the whole population (5,517- and 850-fold, respectively). In the SOT cohort, the liver transplant recipients had the lowest IR (11.1 [7.7-16.3] per 1,000 person-years). The IR of CMV in heart transplant recipients (104.2 [66.4-163.7] per 1,000 person-years) was the highest, followed by multi-organ (72.7 [30.2-175.4]) and kidney transplantation (44.3 [37.7-52.1] per 1,000 person-years) with 8.6, 6.3, and 3.8, respectively, compared to liver transplantation. The lung transplant recipients had a relatively low IR of 17.3 (2.4-123.2) per 1,000 person-years (Table 2 and Figure 1).

### Characteristics of patients with CMV case

Among the 1,586 patients with CMV cases, 283 (17.9%) and 45 (2.8%) patients received SOT and HSCT, respectively. A total of 1,258 (79.3%) patients had no transplantation history (neither SOT nor HSCT). The SOT recipients, HSCT recipients, and non-transplant groups had similar mean age, male percentage, and ICD-10 codes for CMV diagnosis (Table 3). According to transplant organs, the percentage of CMV among total recipients was highest in heart transplantation (25 CMV cases/502 total recipients, 5.0%) followed by multi-organ (6/183, 3.3%), pancreas (2/68, 2.9%), kidney (192/9,381, 2.0%), lung (2/168, 1.2%), and liver transplantation (56/6,066, 0.9%) (Table 3, Figure 1, and Supplementary Table 1). The percentage of SOT recipients aged ≥ 40 years (68.9%) was significantly higher than that in HSCT recipients (55.6%) or non-transplant recipients (60.8%) (*P* = 0.026). The non-transplant population had the significantly higher rates of co-morbid diseases compared to SOT and HSCT recipients (*P* = 0.031). The post-transplantation duration before diagnosis of CMV cases was significantly shorter in HSCT recipients than in SOT recipients (2.2 ± 2.0 vs 3.4 ± 2.7 months, *P* = 0.007) (Table 3).

### All-cause mortalities in patients with CMV cases

**Figure 2:**
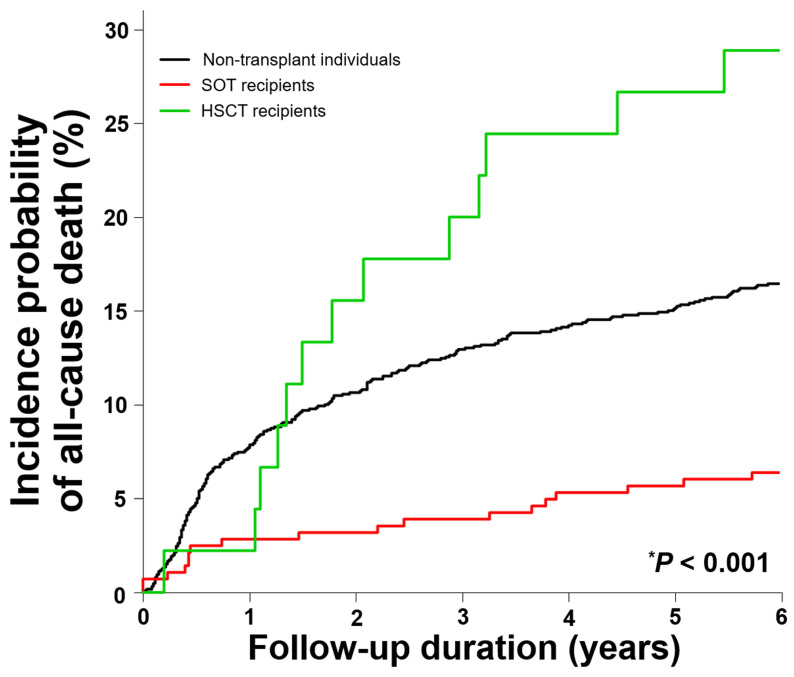
All-cause mortality rates in solid organ transplantation recipients, hematopoietic stem cell transplantation recipients, and non-transplant individuals with CMV disease except CMV syndrome. ^*^Log rank test. Aberrations: HSCT, hematopoietic stem cell transplantation; SOT, solid organ transplantation

The percentage of all-cause death was significantly higher in HSCT recipients (28.9%) than in SOT recipients (6.4%) and non-transplant recipients (16.5%) (*P* < 0.001) (Table 3 and Figure 2). Cox's proportional hazards regression analysis showed that SOT recipients had a significantly lower mortality rate (adjusted IR [95% CI] per 1,000 person-years, 53.2 [33.4-84.8]; adjusted HR [95% CI], 0.4 [0.3-0.7] compared to that of non-transplant individuals with adjusted IR of 130 per 1,000 person-years). But, the adjusted mortality rate in the HSCT recipients (329.4 [190.8-568.7] per 1.000 person-years) was significantly higher with the adjusted HR of 2.4 (1.3-4.0) compared to the non-transplant individuals (log-rank *P* < 0.001). Among transplant organs, only kidney transplantation (33.4 [16.7-67.0] per 1,000 person-years) was associated with a significantly lower mortality rate (adjusted HR of 0.3 [0.1-0.5]) (Table 4).

## Discussion

The new finding in our data from a large entire population-based cohort was that the recipients with post-SOT CMV cases had lower (approximately 60% decrease) all-cause mortality rate than that of non-transplant individuals, although the SOT recipient group had a significantly higher IR (nearly 5,000-fold) of CMV cases after transplantation compared to that of non-transplant individuals. The reduction in mortality rate was distinct in kidney transplant recipients (approximately 75% decrease). The comparison of IR or mortality of CMV cases in transplant recipients and non-transplant group has not been performed yet. Interestingly, the patients with CMV in each group were relatively young with a mean age of 40-44 years. This may be because non-transplant patients with various comorbidities may have a higher risk of adverse outcomes with CMV end-organ disease than SOT recipients, although post-SOT CMV disease could also be attributable to morbidity and mortality 30,31,49. The relatively high all-cause mortality in individuals without transplantation might be related to the various co-morbid illnesses, especially ESRD on dialysis, chronic lung diseases, and HIV-1 infection, and/or the indirect effects of CMV disease, including the risk of infections by other bacteria or fungi 20,30,50.

Litjens et al. recently reported that donor-derived memory-like NKG2C^+^ natural killer cells and Vδ2^neg^γδ T lymphocytes could be expanded by CMV replication, and the terminally differentiated TCR αβ^+^ T lymphocytes with poor alloimmunity, NKG2C gene expression, and resistance of the adaptive immune system driven by interferon could be enhanced during CMV infection 51. These immunological alterations by active CMV replication may be associated with allograft acceptance in kidney, liver, or heart transplant recipients, as well as protection against post-HSCT leukemic relapse in patients with acute myeloid leukemia 27,51-57. Even though further studies are needed to explore more evidence for causal relationships, some studies suggest that CMV infection or disease after transplantation might have beneficial effects to avoid various adverse morbidities or mortality in special recipients 51,58,59. Our epidemiologic results might provide an opportunity to contemplate the large burden of CMV disease in non-transplant settings as well as the potential effect of CMV replication on a poor final outcome in transplant recipients with certain characteristics in actual clinical settings with CMV prevention strategies.

In this analysis, the IR of CMV in the HSCT cohort did not differ considerably from that in the SOT recipients, which may be associated with proper prevention, especially in HSCT recipients with a generally higher risk of adverse effects caused by CMV reactivation. Unlike the prior reports on CMV risk factors, our SOT cohort had a relatively low IR for CMV in lung transplant recipients 30,31. The IR for CMV in lung transplantation may be underestimated due to the exclusion of CMV syndrome in our study. However, all previous studies presenting CMV epidemiology with percentage did not assess IR, taking into consideration the follow-up duration 21-29,31. This first analysis assessing direct comparison of CMV IRs according to transplant organs in a specific cohort showed that liver or lung transplant recipients had lower IRs for CMV, and heart transplant recipients had the highest IRs. The CMV cases in the kidney, liver, and heart transplant recipients occurred at a similar time after SOT (mean of 3.4 months). During the follow-up period, most CMV cases developed in the early post-transplantation period in both SOT and HSCT recipients (mean of 3 and 2 months, respectively, and majority cases within 6 months) (Supplementary Figure 1A and 1B).

This large cohort study is limited by the lack of information on CMV preventive strategies in the SOT and HSCT cohorts. Regardless of the consensus guidelines for CMV prevention in transplant settings, standardized protocols, including dosage or duration of anti-CMV drugs, strategies or surveillance of prevention (especially, application of recent hybrid approaches), cut-off thresholds of CMV viral load, monitoring methods for preemptive therapy, or management of refractory/recurrent/relapsed cases would be quite heterogeneous for each institution or physician 30,35,45,60. This is because sophisticated strategies are based on tailored management in accordance with several risk factors and clinical conditions 30,45. Unfortunately, it was not possible to extract detailed data on CMV preventive strategies from the national database, and this process revealed only a few CMV cases in various subgroups. Additionally, we could not evaluate the IRs of CMV according to the serostatus of donors and recipients, because almost all adult Koreans among general population and transplant recipients are seropositive for CMV 61-63. The small number of CMV cases in the childhood and adolescent groups would assure the extraction of symptomatic CMV cases with the exclusion of congenital CMV infection, CMV mononucleosis, or asymptomatic CMV detection in the total cohort (Supplementary Tables 2 and 3). However, the absence of CMV syndrome in our dataset could underestimate the IR of CMV, especially in the transplant recipients.

Nevertheless, our analysis is the first attempt to report the difference in IR and mortality between non-transplant individuals and SOT or HSCT recipients to assess the burden of CMV cases in clinical practice. Two previous cohort studies evaluated the association of CMV seropositivity with all-cause mortality in the adult general population without transplants, except in the pregnant women group. In 14,153 American adults, CMV seropositive rates were related to higher adjusted all-cause mortality (overall HR of 1.2) 42. Another cohort of 13,000 adults in the UK confirmed that CMV seropositive status (59% seroprevalence) was associated with increased all-cause mortality (adjusted HR of 1.2) 43. However, these studies with small HRs did not compare mortality in intensively immunocompromised patients. Another strength of our study is that we did not use the seroprevalence rates or CMV IgG titers to identify the CMV group. Furthermore, our CMV cohort could exclude asymptomatic CMV detection in blood or body fluids.

## Conclusions

Our large cohort from the entire population-based database showed that non-transplant individuals with CMV, regardless of low incidence of CMV disease, had higher all-cause mortality than SOT recipients. This finding suggests that active CMV replication causing CMV disease may be associated with a potentially large burden because of life-threatening outcomes in other patient groups besides severely immunocompromised transplant recipients.

## Supplementary Material

Supplementary figures and tables.

## Author Contributions

S.H.H and K.H.L designed concept and supervised the study. K.D.H retrieved the cohort data from the large database and performed the statistical analysis. S.G.Y, D.E.K, Y.L and S.H.H summarized the detailed data and wrote the main manuscript text. All authors revised the manuscript critically for important intellectual content and approved the final version.

## Figures and Tables

**Table 1:** Incidence of CMV disease except CMV syndrome in solid organ and hematopoietic stem cell transplantation recipients

Populations		Year					
		2010	2011	2012	2013	2014	2015
**SOT**							
**Total**	Total Pop.	2,360	2,858	3,072	2,971	3,142	3,411
	CMV cases^a^	31	25	45	60	69	53
	Crude IR^b^	1.31 (0.85-1.77)	0.87 (0.53-1.22)	1.46 (1.04-1.89)	2.02 (1.51-2.53)	2.20 (1.68-2.71)	1.55 (1.14-1.97)
	Std. IR^b^	1.34 (0.67-2.01)	1.02 (0.39-1.64)	2.07 (1.07-3.07)	2.97 (1.76-4.17)	3.16 (2.05-4.26)	2.14 (1.26-3.01)
**Kidney**	Total Pop.	1,258	1,604	1,741	1,716	1,775	1,875
	CMV cases^a^	24	19	36	41	42	35
	Crude IR^b^	1.91 (1.15-2.66)	1.18 (0.65-1.71)	2.07 (1.40-2.74)	2.39 (1.67-3.11)	2.37 (1.66-3.07)	1.87 (1.25-2.48)
	Std. IR^b^	1.71 (0.86-2.55)	1.19 (0.37-2.01)	2.44 (1.14-3.73)	7.63 (0-17.08)	2.86 (0.95-4.78)	2.43 (0.51-4.35)
**Liver**	Total Pop.	1,012	1,131	1,191	1,084	1,181	1,307
	CMV cases^a^	4	3	7	12	17	10
	Crude IR^b^	0.40 (0.01-0.78)	0.27 (0-0.57)	0.59 (0.15-1.02)	1.11 (0.48-1.73)	1.44 (0.76-2.12)	0.77 (0.29-1.24)
	Std. IR^b^	0.36 (0-0.89)	0.10 (0-0.22)	1.76 (0-3.8)	3.65 (0.54-6.76)	2.72 (0.65-4.79)	1.19 (0.27-2.10)
**Heart**	Total Pop.	69	92	103	117	112	141
	CMV cases^a^	2	3	2	6	5	8
	Crude IR^b^	2.90 (0-6.96)	3.26 (0-6.96)	1.94 (0-4.65)	5.13 (1.07-9.18)	4.46 (0.58-8.35)	5.67 (1.81-9.54)
	Std. IR^b^	2.12 (0-5.59)	5.94 (0-15.78)	1.32 (0-3.15)	4.87 (0-9.82)	3.16 (0.13-6.18)	5.78 (0.19-11.37)
**Lung**	Total Pop.	18	33	35	43	54	63
	CMV cases^a^	0	0	0	0	2	0
	Crude IR^b^	—	—	—	—	3.70 (0-8.91)	—
	Std. IR^b^	—	—	—	—	8.30 (0-21.66)	—
**Pancreas** **alone**	Total Pop.	25	39	35	60	54	59
	CMV cases^a^	1	1	1	0	3	1
	Crude IR^b^	4.00 (0-12.26)	2.56 (0-7.75)	2.86 (0-8.66)	—	5.56 (0-11.87)	1.69 (0-5.09)
	Std. IR^b^	1.64 (0-4.84)	0.67 (0-1.99)	0.90 (0-2.65)	—	6.99 (0-17.18)	0.52 (0-1.54)
**HSCT**							
	Total Pop.	1,263	1,522	1,639	1,740	1,863	2,179
	CMV cases^a^	4	2	7	5	11	16
	Crude IR^b^	0.32 (0.01-0.63)	0.13 (0-0.31)	0.43 (0.11-0.74)	0.29 (0.04-0.54)	0.59 (0.24-0.94)	0.73 (0.38-1.09)
	Std. IR^b^	0.35 (0-0.73)	0.10 (0-0.24)	0.36 (0.09-0.63)	0.27 (0.03-0.51)	0.49 (0.19-0.78)	0.70 (0.35-1.06)
**Whole population**							
	Total Pop.	50,165,317	50,443,562	50,761,374	51,011,717	51,279,732	51,571,506
	CMV cases^a^	159	206	205	259	359	398
	Crude IR^c^	0.32 (0.27-0.37)	0.41 (0.35-0.46)	0.40 (0.35-0.46)	0.51 (0.45-0.57)	0.70 (0.63-0.77)	0.77 (0.70-0.85)
	Std. IR^c^	0.32 (0.27-0.37)	0.41 (0.35-0.47)	0.40 (0.35-0.46)	0.50 (0.44-0.56)	0.68 (0.61-0.76)	0.75 (0.68-0.82)

Data are expressed as number or IR (95% CI). ^a^Indicate CMV disease except CMV syndrome. ^b^Per 100 person-years. ^c^Per 100,000 person-years. Crude IR means the unadjusted incidence rate. Standardized IRs are adjusted by age and sex using 2010 South Korea Population and Housing Census data. Aberrations: CI, confidence interval; CMV, cytomegalovirus; HSCT, hematopoietic stem cell transplantation; IR, incidence rate; Pop., population; SOT, solid organ transplantation, Std., standardized

**Table 2:** Incidence rates of CMV disease except CMV syndrome in transplant recipients

	Total number	CMV cases^a^	F/U duration (years)	Unadjusted IR	Adjusted IR^b^	Adjusted HR^b^
**Whole population**	50,165,317	1,586	45,494.48	0.005 (0.004-0.006)	0.006 (0.005-0.007)	1 (Reference)
**SOT**	16,368	283	8124.10	33.7 (29.5-38.6)	33.1 (28.8-38.0)	5,517 (4,114-7,600)^c^
Kidney	9,381	192	4645.58	44.3 (37.9-51.9)	44.3 (37.7-52.1)	3.78 (2.54-5.85)
Liver	6,066	56	3024.34	11.5 (7.9-16.7)	11.1 (7.6-16.3)	1 (Reference)
Heart	502	25	246.06	107.4 (68.5-168.4)	104.2 (66.4-163.7)	8.60 (4.71-15.39)
Lung	168	2	83.78	18.2 (2.6-129.4)	17.3 (2.4-123.2)	1.30 (0.07-6.09)
Pancreas alone	68	2	33.87	47.7 (6.7-338.6)	52.9 (7.4-379.9)	3.51 (0.20-16.94)
Multi-organ	183	6	90.47	70.3 (29.3-169.0)	72.7 (30.2-175.4)	6.33 (2.13-15.24)
**HSCT**	10,206	45	1291.82	9.0 (8.0-10.4)	5.1 (4.6-6.1)	850 (657-920)^c^

Incidence rates are expressed as cases as per 1,000 person-years. ^a^Indicate CMV disease except CMV syndrome. ^b^Adjusted for age and sex. ^c^Comparison to the whole population. Aberrations: CMV, cytomegalovirus; F/U, follow up; HR, hazard ratio; IR, incidence rate

**Table 3:** Comparison of characteristics between solid organ, hematopoietic stem cell transplantation recipients, and non-transplant individuals among total 1,586 patients with CMV disease except CMV syndrome

Characteristics	No transplantation (N = 1,258)	SOT (N = 283)	HSCT (N = 45)	*P*-value
**Age, years**	42.1 ± 26.8	43.9 ± 17.0	40.9 ± 16.9	0.501
≥ 40-year-old	765 (60.8)	195 (68.9)	25 (55.6)	0.026
**Sex, male**	699 (55.6)	148 (52.3)	21 (46.7)	0.331
**Co-morbid illness^a^**	636 (50.6)	97 (34.3)	18 (40.0)	0.031
Type 2 DM	375 (29.8)	78 (27.6)	14 (31.1)	0.849
ESRD on dialysis	83 (6.6)	2 (0.7)	1 (2.2)	0.017
Chronic heart diseases	64 (5.1)	8 (2.8)	3 (6.7)	0.125
Chronic liver diseases	38 (3.0)	10 (3.5)	4 (8.9)	0.367
Chronic lung diseases	93 (7.4)	5 (1.8)	1 (2.2)	0.025
HIV-1 infection	20 (1.6)	0 (0)	0 (0)	—
**Transplant organ**				—
Single	—	277 (97.9)	—	
Kidney	—	192 (67.8)	—	
Liver	—	56 (19.8)	—	
Heart	—	25 (8.8)	—	
Lung	—	2 (0.7)	—	
Pancreas alone	—	2 (0.7)	—	
Multi-organ	—	6 (2.1)	—	
**CMV diagnosis^b^**				0.248
B25	3 (0.2)	0 (0)	0 (0)	
B25.0	89 (7.1)	6 (2.1)	0 (0)	
B25.1	101 (8.0)	4 (1.4)	0 (0)	
B25.2	4 (0.3)	0 (0)	0 (0)	
B25.8	465 (37.0)	80 (28.3)	20 (44.4)	
B25.9	596 (47.4)	193 (68.2)	25 (55.6)	
**Intervals between transplantation and CMV cases^c^, months**	—	3.4 ± 2.7	2.2 ± 2.0	0.007
Transplant organs				
Kidney	—	3.4 ± 2.7	—	
Liver	—	3.4 ± 2.8	—	
Heart	—	3.4 ± 2.5	—	
Lung	—	8.1 ± 3.0	—	
Pancreas alone	—	5.0 ± 0.8	—	
Multi-organ	—	2.9 ± 2.3	—	
CMV diagnosis^b^				
B25.0	—	6.2 ± 3.5	—	
B25.1	—	2.0 ± 1.8	—	
B25.8	—	3.9 ± 2.7	2.2 ± 1.2	
B25.9	—	3.2 ± 2.6	2.3 ± 1.9	
**All-cause death**	207 (16.5)	18 (6.4)	13 (28.9)	<0.001

Data are expressed as mean ± standard deviation or number (percentage). ^a^Some patients had several illnesses. ^b^By WHO ICD-10 codes, version 2016 (B25: cytomegaloviral disease, B25.0: cytomegaloviral pneumonitis, B25.1: cytomegaloviral hepatitis, B25.2: cytomegaloviral pancreatitis, B25.8: other cytomegaloviral diseases, B25.9: cytomegaloviral disease, unspecified). ^c^Indicate CMV disease except CMV syndrome. Aberrations: CMV, cytomegalovirus; DM, diabetes mellitus; ESRD, end stage renal disease; HIV-1, human immunodeficiency virus-1; HSCT, hematopoietic stem cell transplantation; f/u, follow-up; ICD-10, International Statistical Classification of Diseases and Related-Health Problems 10th Revision; SOT, solid organ transplantation, WHO, World Health Organization

**Table 4:** All-cause mortality rates in solid organ transplantation recipients, hematopoietic stem cell transplantation recipients, and non-transplant individuals with CMV disease except CMV syndrome in Cox's proportional hazards regression model

	No.	All-cause death	Total f/u Duration (years)	Unadjusted		Adjusted^a^	
				IR (95% CI)	HR (95% CI)	IR (95% CI)	HR (95% CI)
**Non-transplant individuals**	1,258	207	1111.27	186.3 (162.6-213.5)	1 (Reference)	129.9 (107.5-156.8)	1 (Reference)
**SOT**	283	18	271.45	66.3 (41.8-105.2)	0.37 (0.22-0.57)	53.2 (33.4-84.8)	0.42 (0.25-0.67)
Kidney	192	8	186.91	42.8 (21.4-85.6)	0.23 (0.10-0.43)	33.4 (16.7-67.0)	0.25 (0.11-0.48)
Liver	56	8	50.93	157.0 (78.6-314.1)	0.84 (0.38-1.58)	139.7 (69.6-280.4)	1.04 (0.47-1.98)
Heart	25	2	23.61	84.7 (21.2-338.7)	0.45 (0.08-1.40)	69.4 (17.3-277.8)	0.51 (0.09-1.60)
Lung	2	0	—	—	—	—	—
Pancreas alone	2	0	—	—	—	—	—
Multi-organ	6	0	—	—	—	—	—
**HSCT**	45	13	36.91	352.2 (204.5-606.5)	1.81 (0.98-3.04)	329.4 (190.8-568.7)	2.37 (1.28-4.01)

Incidence rates indicate cases as per 1,000 person-years. ^a^Adjusted by age and sex using 2010 South Korea Population and Housing Census data. Aberrations: CI, confidence interval; CMV, cytomegalovirus; f/u, follow-up; HR, hazard ratio; HSCT, hematopoietic stem cell transplantation; IR, incidence rate; No., number; SOT, solid organ transplantation
