# Supplementary material for: The Incidence and Effect of Cytomegalovirus Disease on Mortality in Transplant Recipients and General Population: Real-world Nationwide Cohort Data
Source: Int J Med Sci. 2021 Jul 25;18(14):3333–41. doi: 10.7150/ijms.62621 (PMC8364452; doi:10.7150/ijms.62621)

Supplementary table 1. Clinical characteristics in total 16,368 recipients in solid organ transplantation cohort

Characteristics	Total recipients (N = 16,368)
Age, years	49.4 ± 10.8
20-39-year-old	3,050 (18.6)
40-64-year-old	12,345 (75.4)
≥ 65-year-old	973 (5.9)
Sex, male	10,605 (64.8)
Transplant organ	
Single	16,185 (98.9)
Kidney	9,381 (57.3)
Liver	6,066 (37.1)
Heart	502 (3.1)
Lung	168 (1.0)
Pancreas	68 (0.4)
Multi-organ	183 (1.1)
Kidney & pancreas	150 (0.9)
Kidney & liver	22 (0.1)
Kidney & heart	9 (0.04)
Liver & heart	1 (0.01)
Liver & lung	1 (0.01)

Data are expressed as mean ± standard deviation or number (frequency).

Supplementary table 2. Incidence rates of CMV disease except CMV syndrome by age groups in solid organ transplantation recipients between 2010 and 2015

Age groups	2010			2011			2012			2013			2014			2015		
	No.	Case[a]	IR	No.	Case[a]	IR	No.	Case[a]	IR	No.	Case[a]	IR	No.	Case[a]	IR	No.	Case[a]	IR
**Total**																		
0-9	50	1	2.0	54	1	1.9	48	1	2.1	44	6	13.6	59	9	15.3	59	4	6.8
10-19	71	1	1.4	70	1	1.4	76	4	5.3	77	1	1.3	65	1	1.5	66	1	1.5
20-29	170	2	1.2	177	2	1.1	166	1	0.6	145	5	3.5	166	4	2.4	163	5	3.1
30-39	346	5	1.5	433	3	0.7	395	5	1.3	456	4	0.9	378	11	2.9	403	10	2.5
40-49	659	8	1.2	787	4	0.5	874	14	1.6	751	14	1.9	807	13	1.6	829	7	0.8
50-59	795	7	0.9	1008	12	1.2	1105	16	1.5	1073	17	1.6	1148	21	1.8	1306	21	1.6
60-69	261	7	2.7	314	2	0.6	381	3	0.8	395	13	3.3	485	10	2.1	528	5	1.0
70-79	8	0	—	15	0	—	27	1	3.7	30	0	—	34	0	—	54	0	—
**Male**																		
0-9	25	1	4.0	26	1	3.9	21	1	4.8	21	2	9.5	28	2	7.1	28	1	3.6
10-19	39	1	2.6	38	0	—	45	1	2.2	41	1	2.4	31	0	—	38	1	2.6
20-29	96	1	1.1	94	1	1.1	95	0	—	80	2	2.5	87	1	1.2	74	3	4.1
30-39	197	3	1.5	239	2	0.8	226	3	1.3	263	2	0.8	219	9	4.1	225	6	2.7
40-49	436	5	1.2	526	3	0.6	566	6	1.1	481	6	1.3	514	7	1.4	552	6	1.1
50-59	564	6	1.1	724	5	0.7	745	10	1.3	739	8	1.1	763	8	1.1	926	10	1.1
60-69	176	4	2.3	195	1	0.5	249	0	0	237	9	3.8	324	5	1.54	360	3	0.8
70-79	5	0	—	11	0	—	18	1	5.6	22	0	—	20	0	—	34	0	—

**Female**																		
0-9	25	0	—	28	0	—	27	0	—	23	4	17.4	31	7	22.6	31	3	9.7
10-19	32	0	—	32	1	3.1	31	3	9.7	36	0	—	34	1	2.9	28	0	—
20-29	74	1	1.4	83	1	1.2	71	1	1.4	65	3	4.6	79	3	3.8	89	2	2.3
30-39	149	2	1.3	194	1	0.5	169	2	1.2	193	2	1.1	159	2	1.3	178	4	2.3
40-49	223	3	1.4	261	1	0.4	308	8	2.6	270	8	2.9	293	6	2.1	277	1	0.4
50-59	231	1	0.4	284	7	2.5	360	6	1.7	334	9	2.7	385	13	3.4	380	11	2.9
60-69	85	3	3.5	119	1	0.8	132	3	2.3	158	4	2.5	161	5	3.1	168	2	1.2
70-79	3	0	—	4	0	—	9	0	—	8	0	—	14	0	—	20	0	—

No. and case means the total SOT recipients and CMV cases, respectively. IR indicates the unadjusted crude rates per 100 person-years. [a]Indicate CMV disease except CMV syndrome. Aberrations: CMV, cytomegalovirus; IR, incidence rate; SOT, solid organ transplantation

Supplementary table 3. Incidence rates of CMV disease except CMV syndrome by age groups in hematopoietic stem cell transplantation recipients between 2010 and 2015

Age groups	2010			2011			2012			2013			2014			2015		
	No.	Case[a]	IR	No.	Case[a]	IR	No.	Case[a]	IR	No.	Case[a]	IR	No.	Case[a]	IR	No.	Case[a]	IR
**Total**																		
0-9	70	1	1.4	82	0	—	88	0	—	72	0	—	61	0	—	85	0	—
10-19	182	0	—	243	1	0.4	238	0	—	252	1	0.4	223	0	—	264	2	0.8
20-29	185	0	—	224	0	—	243	3	1.2	245	0	—	255	1	0.4	288	5	1.7
30-39	247	0	—	255	0	—	282	1	0.4	290	1	0.3	309	3	1.0	325	2	0.6
40-49	280	0	—	340	0	—	345	1	0.3	353	1	0.3	375	2	0.5	432	3	0.7
50-59	216	3	1.4	263	1	0.4	311	2	0.6	356	0	—	415	4	1.0	494	1	0.2
60-69	74	0	—	105	0	—	121	0	—	155	2	1.3	207	1	0.5	267	3	1.1
70-79	8	0	—	7	0	—	10	0	—	17	0	—	17	0	—	21	0	—
**Male**																		
0-9	41	0	—	51	0	—	47	0	—	36	0	—	35	0	—	54	0	—
10-19	113	0	—	145	1	0.7	154	0	—	148	0	—	130	0	—	154	0	—
20-29	94	0	—	116	0	—	122	1	0.8	133	0	—	135	0	—	157	3	1.9
30-39	123	0	—	121	0	—	133	1	0.8	141	1	0.7	151	1	0.7	148	2	1.4
40-49	145	0	—	182	0	—	189	1	0.5	200	1	0.5	206	0	—	235	1	0.4
50-59	105	1	1.0	128	0	—	149	0	—	176	0	—	198	2	1.0	253	1	0.4
60-69	42	0	—	56	0	—	57	0	—	72	1	1.4	102	0	—	138	3	2.2
70-79	3	0	—	2	0	—	4	0	—	7	0	—	6	0	—	7	0	—

**Female**																		
0-9	29	1	3.5	31	0	—	41	0	—	36	0	—	26	0	—	31	0	—
10-19	69	0	—	98	0	—	84	0	—	104	1	1.0	93	0	—	110	2	1.8
20-29	91	0	—	108	0	—	121	2	1.7	112	0	—	120	1	0.8	131	2	1.5
30-39	124	0	—	134	0	—	149	0	—	149	0	—	158	2	1.3	177	0	—
40-49	135	0	—	158	0	—	156	0	—	153	0	—	169	2	1.2	197	2	1.0
50-59	111	2	1.8	135	1	0.7	162	2	1.2	180	0	—	217	2	0.9	241	0	—
60-69	32	0	—	49	0	—	64	0	—	83	1	1.2	105	1	1.0	129	0	—
70-79	5	0	—	5	0	—	6	0	—	10	0	—	11	0	—	14	0	—

No. and case means the total HSCT recipients and CMV cases, respectively. IR indicates the unadjusted crude rates per 100 person-years. [a]Indicate CMV disease except CMV syndrome. Aberrations: CMV, cytomegalovirus; HSCT, hematopoietic stem cell transplantation; IR, incidence rate

Supplementary Figure 1. Kaplan-Meier curves for development of CMV disease except CMV syndrome within 1 year after transplantation in solid organ transplant recipients

(A) Total SOT recipients

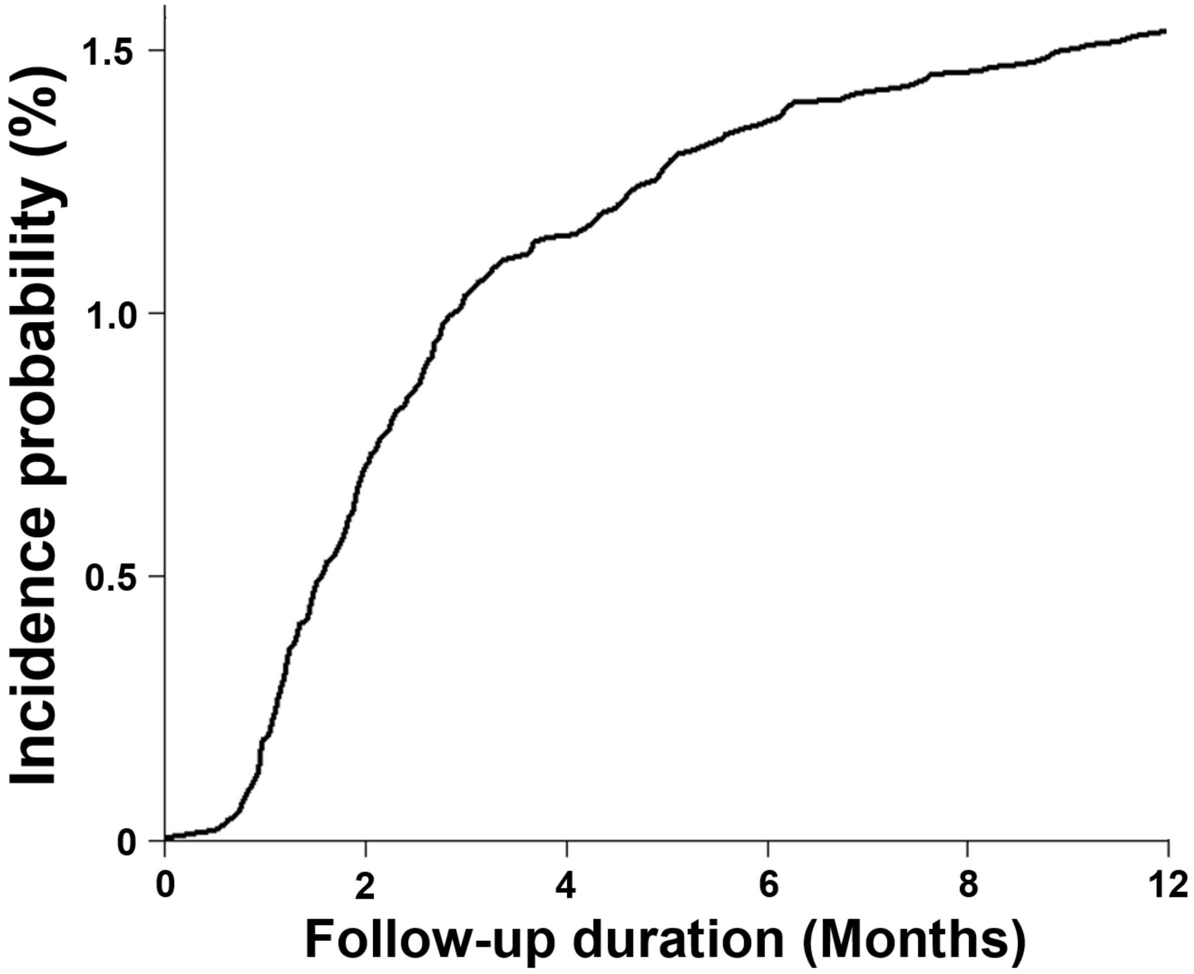

(B) By transplant organs

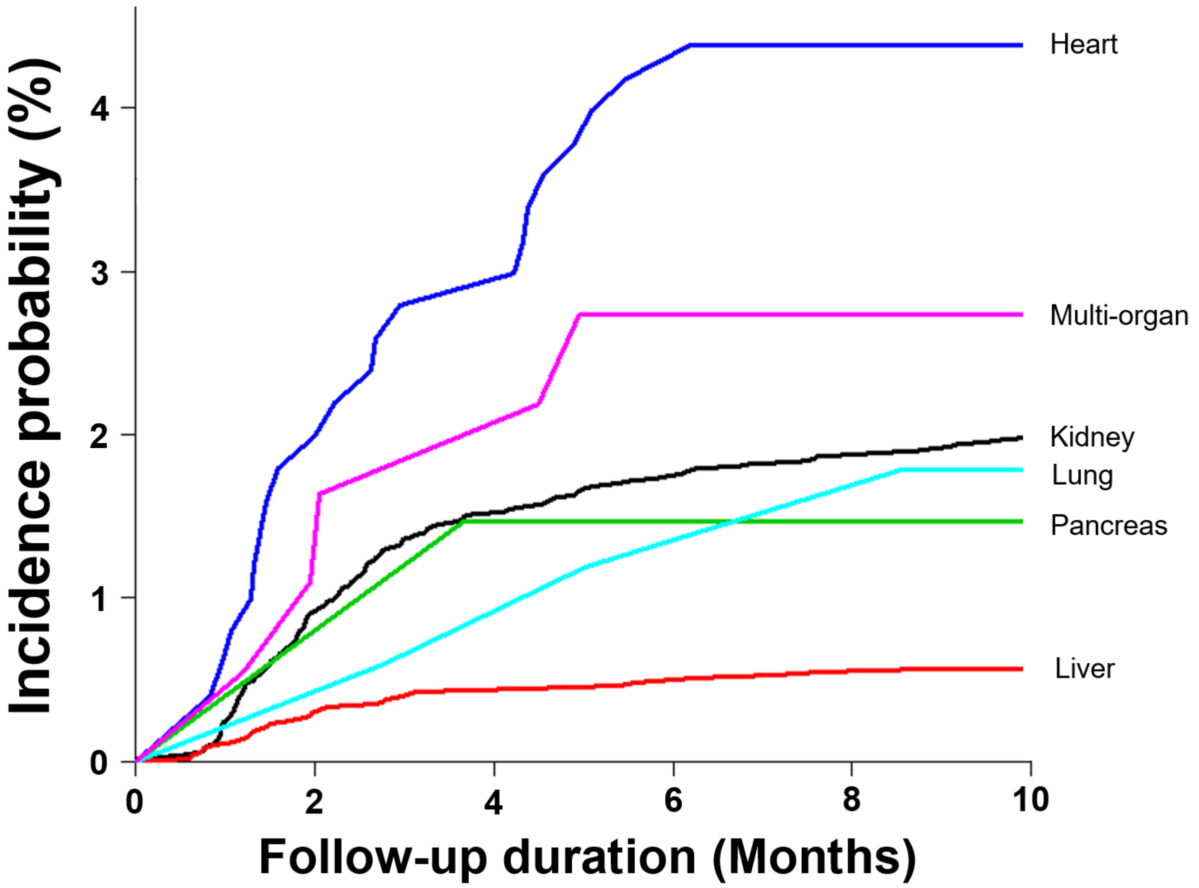

Supplement: Supplementary file 1 — Supplementary figures and tables. [file ijmsv18p3333s1.pdf]
